# Implications of Migration Patterns and Sex Work on Access to Health Services and Key Health Outcomes: A Qualitative Study on Male Migrant Sex Workers in London

**DOI:** 10.1080/19317611.2021.1902893

**Published:** 2021-03-26

**Authors:** Elisa Ruiz-Burga

**Affiliations:** Faculty of Population Health Sciences, University College London, London, UK

**Keywords:** Migration, male sex work, sexual health, health inequalities

## Abstract

**Objectives:**

This paper describes migration toward the UK, sex work involvement, use of health services, and health issues.

**Methods:**

This qualitative study interviewed twenty-five men. The data were analyzed using thematic analysis.

**Results:**

Five main themes emerged: emigration, migration routes, sex work entrance, use of health services, and main health outcomes.

**Conclusions:**

Discrimination and social exclusion were identified before and during migration. Participants had used the NHS system and told positive experiences. They reported STI-HIV, recreational drugs and mental health issues. Findings suggest the influence of migration and sex work on their vulnerabilities and health outcomes.

## Introduction

In the mid-2000s, the economic crisis and the enlargement of the European Union (EU) encouraged a significant increase in migration to the United Kingdom (UK) (Sumption & Vargas-Silva, 2020). The main drivers of immigration were the policies of free movement, wage differentials (Luthra et al., 2014), the British economic growth and structural demand for migrant labor (Czaika & De Haas, 2017). By 2010, the UK hosted one of the largest number of EU and non-EU migrants (Rienzo & Vargas-Silva, 2017), with major concentration in London (Vargas-Silva, 2012). Migration is a public health concern because it can exacerbate vulnerabilities and health inequalities in the countries of destination (Hossin, 2020). In the same way, the impact of immigration on the demand for health services would depend on the health status, health trajectories of immigrants and their routes of entry (Giuntella et al., 2018). Further, migrants’ health can be shaped by their experiences of pre-migration and their experiences during and after migration; work conditions, education, socio-economic status and ethnicity; that influences certain health conditions (Fernández Reino, 2020).

Despite of the growing knowledge of the UK’s foreign-born populations, there is a particular group composed by migrants sex workers (MSW) that has been overlooked. It has been estimated a number of 72,800 sex workers in the UK (House of Commons, 2016), from whom 61% are international migrants (TAMPEP, 2010). The largest origin groups are those from Central and West Europe (66%), Baltic countries (10%), Latin America and Caribbean (10%), and Asia Pacific (7%) (TAMPEP, 2010). The vast majority operates in London with massive presence in indoor settings (97%) (TAMPEP, 2010). Certainly, MSW in other European countries are also characterized as more likely to be migrants, living in large cities, and are considered economic and socially vulnerable (Berg et al., 2015, 2020). The relevance of MSW is based on the fact that they continue to have disproportionately high burdens of STI-HIV infections (Shannon et al., 2018). Studies conducted in London corroborate that migrant male sex workers remain at risk of HIV and other STIs as gonorrhea (Sethi et al., 2006) and chlamydia (Mc Grath-Lone et al., 2014). It is important to note that there is gap of knowledge about other frequent health issues that male migrants engaged in sex work can report as part of their men's health. Furthermore, migrant sex workers are greatly exposed to discrimination, social exclusion and social inequalities to access health care services, due to the convergence of oppressive rules against migration and sex work (TAMPEP, 2019). Yet, in many cases the access to specialized health care for body modifications as a need for transgender/transsexual persons is neglected exposing them to illicit procedures that can risk their safety (Padilla et al., 2016). This paper focus on the drivers of international migration of male sex workers, connections between their trajectories of migration toward the UK and their insertion into sex work, access to UK health services, and main health outcomes reported.

## Materials and methods

### Study design and participants

This qualitative study was conducted from May 2013 to August Berg et al. (2015). The study enrolled biological men, aged 18 and over, non-UK born, living in the UK for at least a year, and who had worked or were still working as sex workers. This study was revised and approved by three local Ethic Committees. They decided that participants should be only contacted through local health services and health projects. This decision aimed to guarantee a safe environment to recruit and interview participants. In this manner, participants were primarily recruited from a Sexual Health Clinic and local projects that were providing health services and counseling to male and female sex workers in London. Doctors, nurses and health workers collaborated with the recruitment of participants providing the participant information sheet (PIS) to potential participants following the aforementioned inclusion criteria. Those who were interested in taking part of this study freely contacted the researcher to receive additional information and/or to agree the date and time for the interview.

### Procedures

The interview guide was developed based on relevant literature and the first-hand information provided by gatekeepers working in projects dedicated to sex workers in London. Overall, twenty-five participants contributed with the study. Written informed consent was obtained from all of them.

### Qualitative methods, data collection and data analysis

Face-to-face in-depth interviews were conducted in suitable locations following the indications of the Ethical Committees. The interviews were conducted until data saturation was reached. The audio recordings were verbatim transcribed, the data stored and organized using ATLAS.ti v 8·0, and analyzed following thematic analysis (Braun & Clarke, 2006). The analysis was centered on the reasons to leave the home countries, the trajectories of migration toward the UK, engagement in sex work, working conditions in the UK, the use of health services, and health outcomes frequently reported.

## Results

### Characteristics of the participants

A ‘convenience’ sampling method provided a heterogenous group of twenty-five men mostly composed by Latin-Americans and Europeans. The mean age of participants was 33 years, 76% self-reported as gay, and 48% had only achieved basic levels of education. The average age of migration was 24, and the number of years doing sex work was about 6 (See Table 1). The analysis of the data provides main themes (See Table 2) that are explained below with illustrative quotes.

**Table 1.** Characteristics of participants.

	**Total (*n* = 25)**
**Home-country**	
Brazil	10 (40%)
Colombia	02 (8%)
Bulgaria	02 (8%)
Spain	06 (24%)
Italy	02 (8%)
Portugal	01 (4%)
Latvia	01 (4%)
Nigeria	01 (4%)
	
Age (mean [range], years)	33 (24–44)
	
**Trajectories of immigration**	
Direct migration in the UK	14 (56 %)
Multi-stage migration toward the UK	11 (44%)
	
Age at emigration (mean [range], years)	24 (13–40)
Time in the UK (median [range], years)	06 (1–23)
	
**Entrance into sex work**	
Age (median [range], years)	27 (14–41)
Time in sex work (mean [range], years)	06 (1–16)
	
**Level of education achieved**	
Primary	02 (8%)
Secondary	10 (40%)
Further education	02 (8%)
Higher Education	11 (44%)
	
**Sexual orientation**	
Gay	19 (76%)
Bisexual	06 (24%)
Heterosexual	0
	
**Currently using recreational drugs at work**	
Yes	15 (60%)
No	10 (40 %)
	
**Report of STIs**	22/ (88%)
Syphilis	12 (48%)
Gonorrhea	15 (60%)
Chlamydia	16 (64%)
Herpes	02 (8%)
Genital warts	03 (12%)
HIV	03 (12%)
Receiving ART	03 (12%)
	
**Mental health issues**	
Low self-esteem	09 (36%)
Depression	15 (60%)

**Table 2.** Interview script and main themes.

Themes	Questions
Leaving the country of origin	How long have you been in the UK? Have you been in other countries before to come to the UK? If yes, can you tell me which countries? [explore for how long/what they did there] Would you tell me about the first time that you left your country? [explore: reasons to migrate/ stories of migration in his family or social context/ family/education/employment]
Migration toward the UK	Why did you decide to come to the UK/London? [explore: reasons to select this country and it occurred] What happened when did you arrive to the UK? [explore their experiences about language/culture/ employment/socio-economic boundaries] Was it easy for you to adapt to this country? If not, what were the difficulties that you experienced at that time? How long did that go on? What made you stay in London? When did you decide to sell sex for money? Why did you make that decision? [explore what was happening in his life at that time/who told him] Have you ever exchanged sex for money or goods in your country? If yes, would you tell me about that
Sex work in London	How many clients did you have last week/month? Do you sell sex to women? /men? /couples? [explore main ethnicity /types of services requested] What are the services that you are willing to do? [explore types, description, prices] What are the services that you are not willing to do? Why? Please explain me about your clients: how/where you contact them? Do you use advertisements to offer your services? If so, where? How you describe yourself in your advertisements?
The use of health services in the UK	Have you ever been sick in this country? If so, what have been your main health problems? [explore health issues in general] do you want to share with me these experiences? [explore what were routes that he followed - doctor?/ self-medication?/friends or family advice?] Are you registered with a GP in the UK? Have you ever used this service? Please tell me about this experience. Have you ever felt discriminated in the NHS services because your migration status? /sexual preferences? / your work as an escort/ethnicity? If so, do you want to share with me these experiences Have you ever disclosed to your GP your work as an escort? If yes, what was the staff’s reaction? Why? Do you think is this important? Why? If not, why?
Sexual health issues reported	Have you heard about sexually transmitted infections?/ HIV? What did you hear? Are you worried about them? Why? Have you ever take any STI test? When/Which/ Why/ would you tell me what was your result? What happened after? Have you ever take HIV test? When/ Why/Do you tell me what was your result? [explore in case positive if he is receiving treatment] Have you ever been in a sexual health clinic in the UK? Would you tell me your experience?

### Leaving the country of origin

The most predominant narratives indicate that leaving the country of origin emerged from the awareness of hard living conditions and economic deprivation of their families. Many, if not most, of stories describe fatherless families. These statements suggest that participants felt responsible to provide for their mothers and families, and consequently, had to drop out of school causing them a sense frustration:

when I was 20, I only had the studies of an 8-years-old boy and this caused me lots of uncertainty, lots of frustration, social anxiety (A, 36, Brazil)

A second, and frequent, reason leaving the country of origin was poor employment prospects. The majority of narratives show that participants were engaged in skilled and unskilled jobs, but decided to leave their countries feeling disappointment at their salaries, lack of opportunities for self-development and professional improvement, and absence of appreciation in their work places:

He told me - if you are not happy you can go because there are lots of people who can stay in your place, you know, you have to be grateful (Le, 26, Brazil)

Another, and not so often acknowledged, reason for leaving their countries of origin from their countries of birth was related to the awareness of same-sexual orientation. This is consistent with the fact that many interviewees self-reported as gay or bisexuals. Some reported female partners to hide their sexual orientation from their families, whereas those who were open about their sexualities experienced homophobia and violence. Some participants *‘ran away*’ from home several times and two attempted suicide:

When I got in gay life I thought I cannot stay here because I had a normal life, I had a partner; I had a normal life (Eva, 41, Colombia)

She [mother] felt ashamed of me, but she was unable to chat with me, and explain to me what was happening because until then I didn’t know what was wrong with me (A, 36, Brazil)

Equally important to note, is that some participants engaged in sex work before leaving their countries of origin; few of them at very young age, as they had to provide for their families. Their accounts associated their initiation in sex work with the use of drugs sex and dropping out of school:

I helped my mother because my family was always poor and the opportunity to have money and help my family was the reason that motivated me to be an escort (Ro, 28, Spain)

sex and drugs became big part of my life, the way that threw out my life, so sex it is a way out, sex industry helped me when I was run out of ways out (B, 30, Portugal)

### Migration toward the UK

This theme describes two different trajectories of migration to the UK and engagement in sex work. The first was followed mostly by Latin-Americans, who experienced multi-stage migration. They migrated from their countries of birth to one or more European countries before coming to the UK. Family members, friends or acquaintances influenced the selection of the first receiving countries. They provided information about migration policies, characteristics of the receiving country, routes of entry and accommodation. It is worth noting that several of these interviewees started selling sex in the first receiving countries motivated by the lack of job opportunities or experiencing hard working and living conditions:

every time that I searched for work, immediately people saw me they realized I was gay and they didn’t give me the job (Gen, 39, Bulgaria)

The circumstances of some interviewees were aggravated because their illegal migration status:

when you don’t have anything to lose, you just go. I think it was that happened to me, I never had anything to lose, just life. (Le, 26, Brazil)

After a number of years, participants who were operating as sex workers in the first receiving country decided to migrate to the UK because they were experiencing language barriers, unsafe sex work environments, and economic crisis:

I didn’t understand things clearly because it was the first time for me doing this, you know, for me was like, he gives me money and I fuck him, and no problem, you know, but the problem started when I have to understand what exactly involves this work (E, 26, Bulgaria)

he said – ‘I worked too much I am going to a hotel because it is difficult to sleep’, and he was gone and after three days the police called – ‘you know this guy, he is dead’ (Le, 26, Brazil)

The majority of discourses show that these participants came to the UK attracted to a wealthier economy, expectations of a more profitable sex market in London, better work conditions, and they knew some English:

Things are quieter here, people here are more discreet and police here is less scary (Gen, 39, Bulgaria)

There are more people who are better off… More clients, more calls, I have more clients that I have in Spain and I can charge more money of course. In Spain, I charged 50 Euros, here I can charge £100 (C, 42, Spain)

I decided to come here because I speak a bit of English and, of course, there are other countries which are better as Germany, Switzerland, France, but the problem was the language (Eva, 42, Colombia)

Differently from their first migration, moving into the UK was principally facilitated by friends and acquaintances who were already part of the local sex work network.

The second trajectory was essentially followed by Europeans who migrated from their countries of birth directly in the UK. Some of them had started to sell sex in their home countries. Most of the narratives indicate their intention of taking on other types of jobs in the UK, however, language barriers and hard working conditions pushed them back into sex work:

We were looking for cleaning job or anything, we were looking for any random job, but we could not get anything, honestly we did not get anything (Jo, 30, Spain)

It should be underlined that several interviewees who migrated directly in the UK, and others who had previously migrated to other European countries, sold sex for the first time in London. Similar to previous experiences, the majority of the discourses indicate that they had difficulties in getting a job, feeling depressed working in unskilled jobs or because their illegal status:

I couldn’t speak with people, I didn’t like that I had to work like as cleaner and then doing the washing up, I found it kind of humiliating because I used to work in a better job in Brazil […] I started to feel depressed. (G, 40, Brazil)

It is important to highlight that few participants traveled to the UK with the intention of selling sex for the first time, received information from peers:

You have brown hair and dark skin, that attract Europeans, why don’t you try to move to London? It is very good spot, go there, stay for a while and work in this, make some money and you will have a good time (Gil, 29, Brazil)

I came directly to [name of the brothel removed] because a guy told me about this and he worked there when he was studying at the university (D, 28, Latvia)

Similar to these narratives, there were others that connected the participants' involvement in sex work with partners, friends, or acquaintances who were already sex workers. These peers provided them with information and guided their entrance. None of our participants told that they were forced by peers to get involved in sex work.

### Sex work in London

Participants had been living in the UK for about 6 years. While the great majority had the right to stay in the country as European citizen, UK same-sex partner or student visa; only few admitted their illegal status. At the time of the interview, a major proportion was living in a flat as sole tenants, and very few were sharing the accommodation. Likewise, the whole group was working as independent internet-based escorts in London. Some common - though not majority - work practices were identified, such as working in other fields (including adult films); combining sex work with other jobs such hair dresser, gym personal trainer, and traveling within the country and overseas to provide sexual services. Participants were operating individually or in couples doing in-calls and out-calls, and reported about 8 clients per week. In addition, all of them offered services to men and several – regardless their sexual orientation, provide services to women as part of ‘couple services’. In terms of their personal lives, almost half told having a 'formal' partner (romantic relationship) at the time of the study, but only few had disclosed their sex work with them.

### The use of health services in the UK and sexual health issues reported

Except participants who informed about visa issues, almost all were registered with a general practitioner (GP) for primary healthcare, and had obtained referrals to other the health services (NHS) for a variety of health problems:

I got everything. For example I had surgery on my knees, trying to stop smoke, no very successfully. So, yes, I have been here for ten years and I have used NHS every time that I needed. B, 30, Portugal

I see my GP when I have caught a flu, headache, when I need my hormones… K, 36, Spain

The majority of narratives suggest that participants did not disclose their occupation as escorts to their GP, mostly because they chose to share this information with health professionals of sexual health clinics:

No, because I can go to Paddington for my work, to do my sexual health checks while the GP is more when I get sick, or I got a flu Ro, 28, Spain

…I saw my GP maybe 2 or 3 times in these three years. So I don’t see her quite often. I think what I should give her is only information about my general health, but my sexual health I will keep that with [name of the nurse removed]. D, 28, Latvia

While the majority of accounts indicate that visiting the GP was not very frequent (once or twice a year), interviewees were inclined to visit sexual clinics more often (every 3 or 6 months) for testing, and receiving condoms and lubricants. The majority of discourses indicate that they were very satisfied with the treatment received in the health services:

I think this is one of the best things that England has is the NHS service; it is there if you need it. Every time that I wanted it, it was there, waiting for me. When I got depressed and I was scared because I don’t want to be sectioned because of my previous suicide attempts, I never did a booking for that B, 30, Portugal

I have been always well treated. I was very good impressed the first time that I used the services, they helped me a lot, they wrote letters to receive treatment, I feel very comfortable. Here is very different from Spain, when I was there I avoid visiting health services because the way that they treat me…. A, 36, Brazil

However, there were other narratives that suggest dissatisfaction and disappointment:

I came to the dental service once here and the dentists put something and burn my mouth and in 15 days I couldn’t work. I was lucky because I had a client at that time, who was a doctor, and he gave antibiotics. Le, 26, Brazil

Sometimes happen that people look at me in a funny way. They say – he doesn’t speak English or they say that we go there to get advantage because the health service is free. Once I was in the health center and one of the secretaries treat me bad because I filled a form requesting to receive medication for my thyroid problem Eva, 41, Colombia

In regards of sexual health outcomes, the diagnosis of STIs was commonplace. The most frequent STIs were chlamydia, gonorrhea and syphilis (See table). Some of these participants told that the first time that were diagnosed with these infections, they were living in other countries.:

…the first time that I had syphilis was when I was 15, I was in Brazil I didn’t know what was it […] then I had gonorrhea, and then I had chlamydia but here, I didn’t know it because I never heard about it in Spain or South America, I have also got herpes… A, 36, Brazil

There were few interviewees who had acquired HIV and were receiving ART treatment from local health services. Overall, their perspective of the UK health services was positive, specially their patient-provider relationship:

my first doctor he was amazing, he was extremely strong and very well supporter. He was very open to try to make me understand that my English was no fluent to understand all the things […]he wasn’t judgmental, he was very open saying – ‘listen he could not do this treatment and drink alcohol because it is too much for your kidney… Geo, 37, Brazil

It is good service, in fact it is quicker than Barcelona. I went there the receptionist gave me a card with a number and after two or three people, they finally saw me […] I have a positive perspective of life, I will not throw myself on the rail, what should I do that? I have hopes that maybe in 5 or 7 years they found a treatment which eliminates the virus and I can stop taking medication In, 36, Spain

Finally, recreational drug use was common among participants. Some were using them at work as part of an overnight service called ‘chem sex’ that was mostly described as polydrug sessions. Other frequent issues informed were insomnia (for more than two days), paranoid behavior, loss of appetite, and depression. They associated these conditions to their drugs' consumption. They also reported low self-esteem, depression and five informed suicide attempts:

I am taking tablets for depression, especially when I don’t feel well I don’t want to have sex, I cannot get excited or anything, so I try to avoid the tablets when I need to work. Also happened once that I got aggressive with this guy (B, 30, Portugal)

## Discussion

In this study, the experiences of 25 migrant male sex workers were explored using in-depth interviews followed by thematic analysis. The findings show that emigration was provoked by hard living conditions and economic deprivation that participants and their families experienced. As others have reported (Vogel, 2009; Weine et al., 2013), our interviewees decided to leave their home countries feeling responsible to financially support their mothers and families. Moreover, this role of economic providers drove some interviewees into sex work at very young age, the use of drugs, and to abandon the school. This finding insinuates a negative impact of poverty on the socialization of boys with the role of provider, particularly in fatherless homes; which is consistent with previous characterization of young male sex workers (Cusick, 2002). Likewise, participants emigrated to look for better employment opportunities as they were experiencing disappointment at their salaries, lack of opportunities for self-development, professional improvement, and absence of appreciation in their work places. These findings support published observations on young migrant sex workers that confirm that low-paid jobs not only provide poor incomes (Van Blerk, 2008), but also disempower and devalue workers with a negative impact on their autonomy, personal development, and self-esteem (Gough et al., 2006). In this manner, this study associates emigration with poverty, unfavorable living and working conditions (Molnar, 2011 ; Rodriguez et al., 2012). In addition, our participants indicated awareness of their same-sex sexual orientation and homophobia as equally important reasons for their emigration. These factors are consistent with prior research on migration of gay, bisexual, and transgender groups (Bhugra et al., 2011) and male sex workers (Mai, 2009; Vogel, 2009). These findings can be explained through the lenses of the minority stress theory (Meyer, 2003) that describes the chronic stress that LGBTQ (lesbian, gay, bisexual, transgender, and queer) groups living in hostile and heterosexist environments, because they are stigmatized provoking internalized homophobia with a negative impact in their social and egalitarian integration. To this extent, the stories of our participants exposed a wide variety of social determinants that may have impacted on their physical and mental health in the stage of pre-migration.

The findings show that the engagement in sex work among our participants occurred distinctly. While several Europeans started sex work in their home country, Latin-Americans mostly engaged in this occupation when they left their country of origin. In general, participants decided to start selling sex when they were struggling to get employed or were experiencing poor working conditions cause by the lack of educational skills. This aspect corroborates claims (Biello et al., 2017) that a background characterized by poverty and low educational achievement restrict migrant sex workers to get better paid jobs, which makes arguable looking for better employment opportunities as one of the main reasons for emigration (Mimiaga et al., 2009; Wirtz et al., 2014). On this basis, sex work was perceived by participants, as a more accessible and rewarding job that could improve their standard of living (Mai, 2013). Many of our participants reinforced their entrance in sex work due to the necessity to financially support their families. In this light, our findings contradicts early claims (Earls & David, 1989) that family background may be less important than other factors driving the entrance in male sex work, and rather supports its relevance (Mai, 2012). Equally important to note, is that our findings show concordance with authors (Srivastava & Goldbach, 2017) that associate friends and partners to the entrance in sex work. The interviewees connected their stories of selling sex for the first time with partners, friends or acquaintances already working as sex workers. So far, none of them felt coerced to take this decision (Biello et al., 2017). As many of the participants engaged in sex work in the first receiving countries, they eventually decided to immigrate again to other European countries. They left those countries because were experiencing language barriers, unsafe sex work settings, and economic recession. Our participants came to the UK attracted by its strong and stable economy, advantageous characteristics of the sex market, and the presence of friends or acquaintances linked to sex work that helped them to settle in London.

Regarding the use of health services in the UK, almost all of the participants had used the NHS system at least once. They were registered with General Practitioner for primary care, had frequently used specialized sexual health clinics, and obtained referrals to hospitals for several medical reasons (e.g. surgeries, hormones, dentist). In this manner, some of our participants confirm the use of specialized health care for body modifications (e.g. prescription of female hormones) (Padilla et al., 2016). Yet, as other authors (Benoit et al., 2019) argue, the interaction with health providers was described by our participants as comfortable, nonjudgmental and supportive; even though they did not disclose their occupation as sex workers to their GP (Bungay et al., 2013). They thought it was no need for the GP to know their occupation, and conversely, they felt more appropriate to disclose this information in the sexual clinics when they request STI-HIV screening, condoms and lubricants because of their job. It may be surmised, then, that identity disclosure did not trigger mental distress in environments perceived as free from violence or discrimination that secure their rights (WHO, 2012).

In addition, this study found that participants reported a wide variety of health issues that corroborates their high-risk of STI-HIV infections (Mc Grath-Lone et al., 2014), high consumption of recreational drugs (Mimiaga et al., 2009) and mental health problems (Poliah & Paruk, 2017). Some of these issues were diagnosed and treated in previous receiving countries. Most of these issues are associated to their occupation as escorts, but also to their multiple stigmatized identities that expose them to health disparities (Logie et al., 2012). Recent contributions assert that sexual minorities such as gay and bisexual men, sex workers, and immigrants cope with internalized stigma that can significantly impact on their mental health and sexual behavior outcomes (Logie et al., 2012; Rendina et al., 2017). Thereby, sexual orientation, gender identity and a stigmatized occupation as sex work are considered significant social determinants of immigrant health (Fox et al., 2020). In addition, it is important to point out that participants' involvement in sex work occurred in different countries and stages of migration, they experienced various sex work settings and work conditions that could affect their physical and mental health. Likewise, their access to health services along their trajectories could be dissimilar as only a minority of EU countries provide to immigrants the same access to health care services (Castaneda, 2013) or they don’t know how to access these services (McDaid et al., 2010). These findings support claims that discrimination, social exclusion and health inequalities manifested through disparities in the access to health services can have detrimental consequences in immigrants' health and increase their exposure to STI and HIV (Kismödi et al., 2017).

Consequently, the findings of this study acknowledge the potential impact of the overlap of migration and sex work on the health outcomes of vulnerable sexual minorities. It was found that discrimination, social exclusion and economic deprivation were significant health determinants. Our findings emphasize the relevance of acknowledging people's decisions on their sexual orientation, gender identity and free involvement in sex work as part of their sexual rights, which are part of human rights. Further, the findings advocate for the decriminalization of sex work as there is evidence of a positive impact on sex workers’ access to health services, occupational health and safety programs (WHO, 2015, 2017). Yet, in England sex work is not illegal and the government has made great efforts to implement specialized health services accessible for this population, evidence still shows poor health outcomes related to STI-HIV, drug's consumption and mental health issues. This aspect calls for a thorough evaluation of these health programs to improve sex workers' health from the perspective of occupational health, but more importantly that considers the nuance experiences of male migrants.

### Strengths and limitations

The findings of this study should be assessed within the limitations. Having only recruited participants from sexual health clinic and health projects in London, the perspective of migrants who do not attend these health services has been missed. Although this limitation, this is one of the few studies on male migrants sex workers that captures their health outcomes and health-seeking experiences in the UK. Our sample reflects the diversity of male migrants involved in sex work and provided rich qualitative data on the connections between reasons for migration, trajectories toward the UK, their insertion in sex work and potential social determinants and inequalities that may have influenced their health outcomes.

### Conclusions

The findings of this study suggest that despite reports showing that migrants are healthier, on average, than the UK born (Fernández Reino, 2020); some groups such as sex workers might have poor health outcomes. Specifically, our findings suggest that emigration was a response to precariousness, oppressive conditions and homophobia, which validates economic and non-economic reasons as equally important factors of migration. Then, it can be suggested that participants were exposed to social exclusion and discrimination since the pre-migration stage. Migration toward the UK followed two distinctive trajectories according to the country of birth of participants. While, Europeans migrated directly from their home countries, Latin Americans had previously migrated to other European countries. Consistently, for the large portion of participants the engagement in sex work occurred in different stages of these routes, when they were struggling to obtain a job or were experiencing poor working conditions. Participants who become sex workers in the first host countries were attracted to the UK wealthier economy, advantageous characteristics of the local sex market, and the presence of friends and acquaintances connected to sex work. Finally, it was found that almost all participants had used the UK health system for several health reasons, and the great majority had a positive perspective of the treatment received. The most frequent health outcome reported were STI-HIV infections, many of which were diagnosed before their migration in the UK. They also frequently reported the use of recreational drugs and mental health problems. Overall, the findings suggest that discrimination and social exclusion before and during the process of migration and involvement in sex work may have influenced the health outcomes of our participants.
